# Supplementary figures and images for: Evidence of Müller Glia Conversion Into Retina Ganglion Cells Using Neurogenin2
Source: Front Cell Neurosci. 2018 Nov 12;12:410. doi: 10.3389/fncel.2018.00410 (PMC6240664; doi:10.3389/fncel.2018.00410)

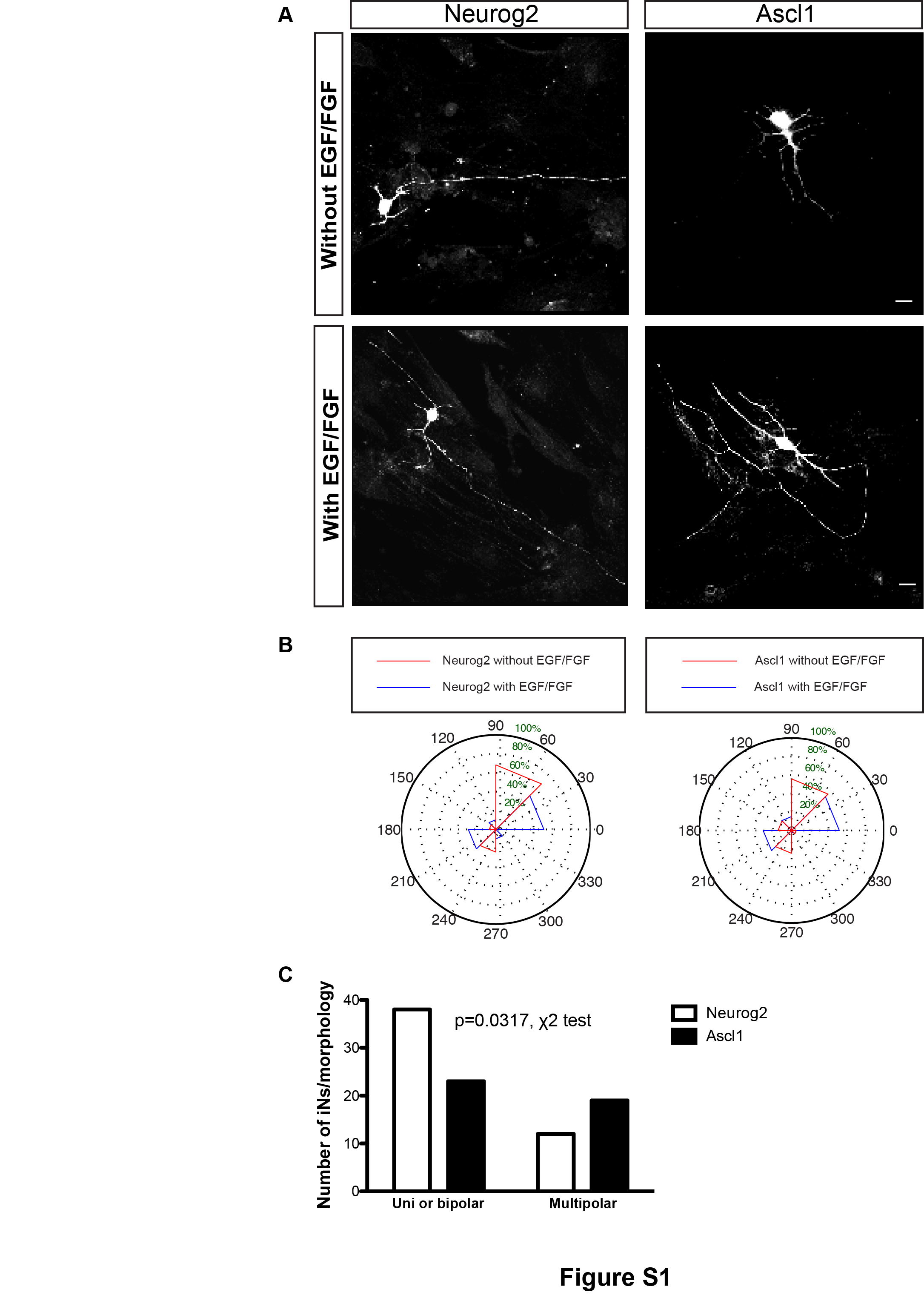

Supplement: Figure S1 — Polarized morphologies are more common in Neurog2- than Ascl1-iNs. (A) Morphologies of iNs derived from MGCs nucleofected with either Neurog2 or Ascl1. (B) Cartesian plot representing the orientations of primary dendrites in iNs generated in different conditions (Neurog2 without GF = 31 iNs; Neurog2 with GF = 19 iNs; Ascl1 without GF = 21 iNs; Ascl1 with GF = 26 iNs). (C) Frequencies of iNs showing unipolar, bipolar, or multipolar morphologies according to the TF used (Neurog2 = 50 iNs; Asc1 = 47 iNs). Observe the higher number of uni/bipolar among Neurog2-induced neurons (p < 0.05; Chi-square test). [file Image_1.JPEG]

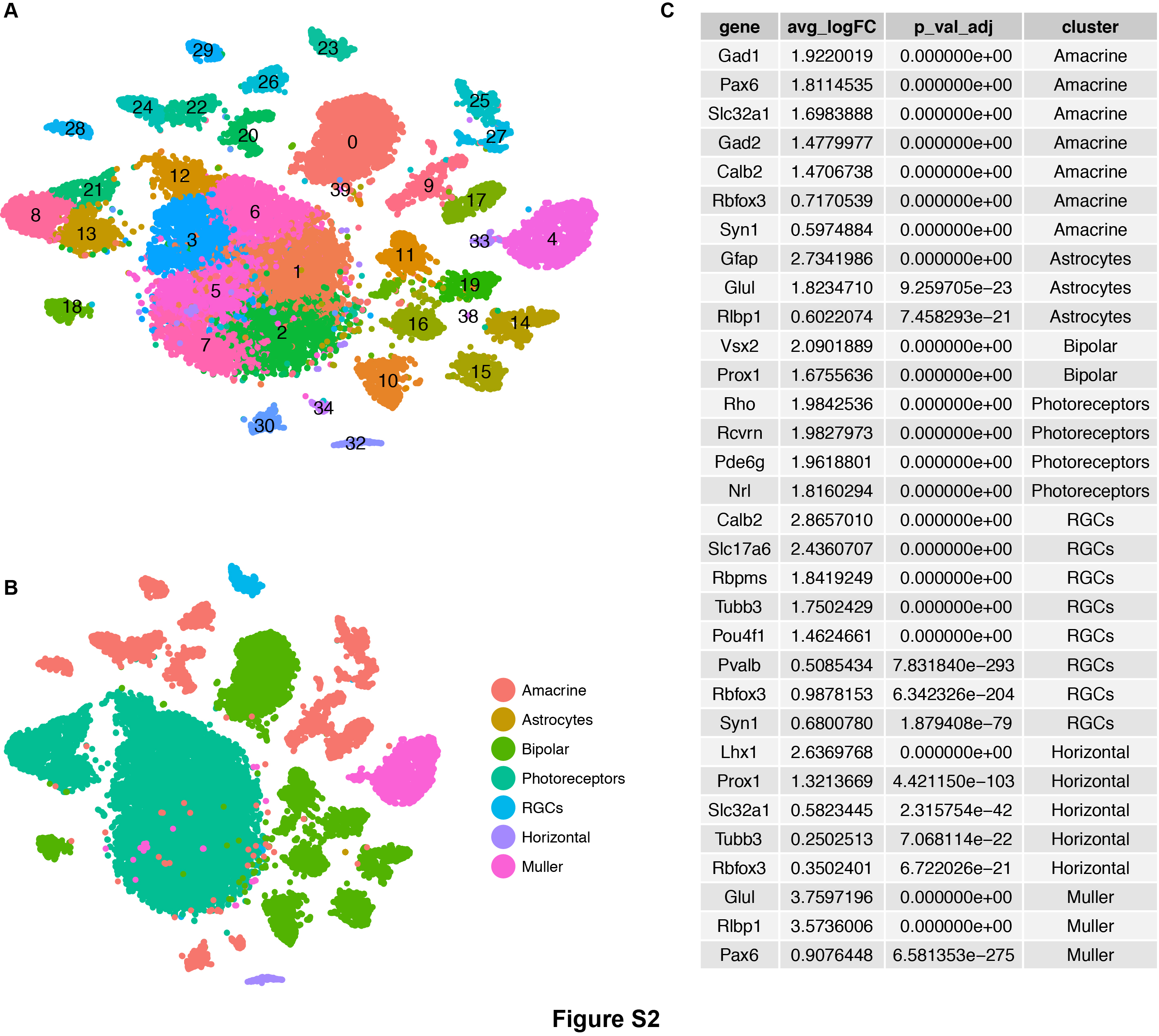

Supplement: Figure S2 — Identification of major retina cell types using single-cell transcriptomes. (A) Clustering of 21,176 single-cell expression profiles into 39 retinal cell populations using available dataset (Macosko et al., 2015). The plot shows a two-dimensional representation (tSNE) of global gene expression relationships among 21,176 cells. (B) Annotation of the same cells into the 7 major retinal cell types, according to the expression of cell-type specific genes. (C) List of some differently expressed genes used to classify the 7 major retina cell types and also used to identify the phenotypes of iNs in this study (RT-qPCR and immunocytochemistry). [file Image_2.JPEG]

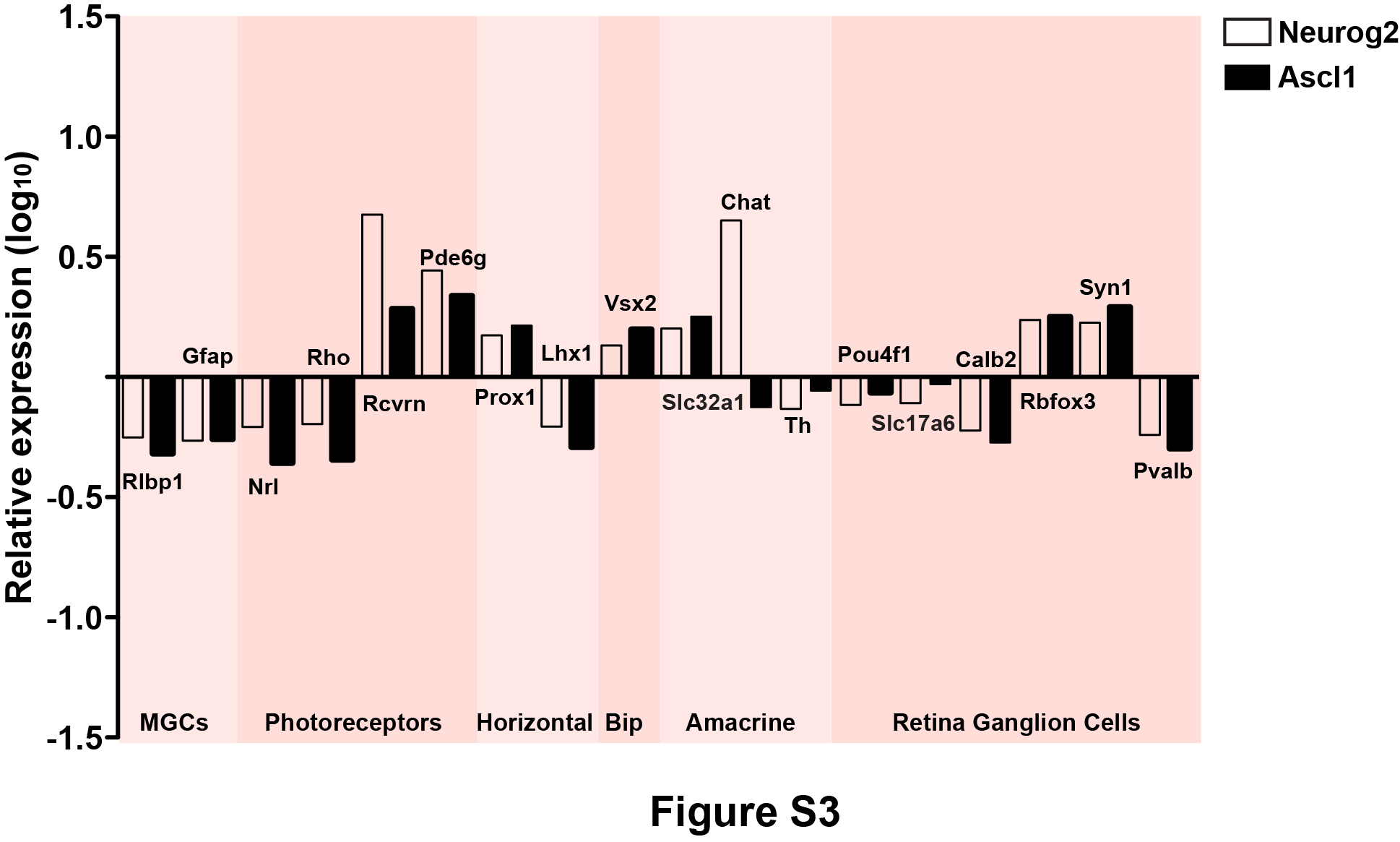

Supplement: Figure S3 — Gene expression in cerebellar astroglia cells nucleofected with Neurog2 or Asc1. Graphic showing the relative expression levels (log10) of genes used to identify presumptive retina cell phenotypes (Figure 5) cerebellar astroglia cell cultures nucleofected with either Neurog2 (white bars) or Ascl1 (black bars). Observe that genes commonly observed in cerebellar neurons (Prox1, Vsx2, Slc32a1, Chat, Rbfox3, and Syn1) are upregulated, whereas genes whose expression is restricted to retinal neurons (Nrl, Rho, Pou4f1, Slc17a6) are not up regulated in cerebellar astroglia-derived iNs. [file Image_3.JPEG]

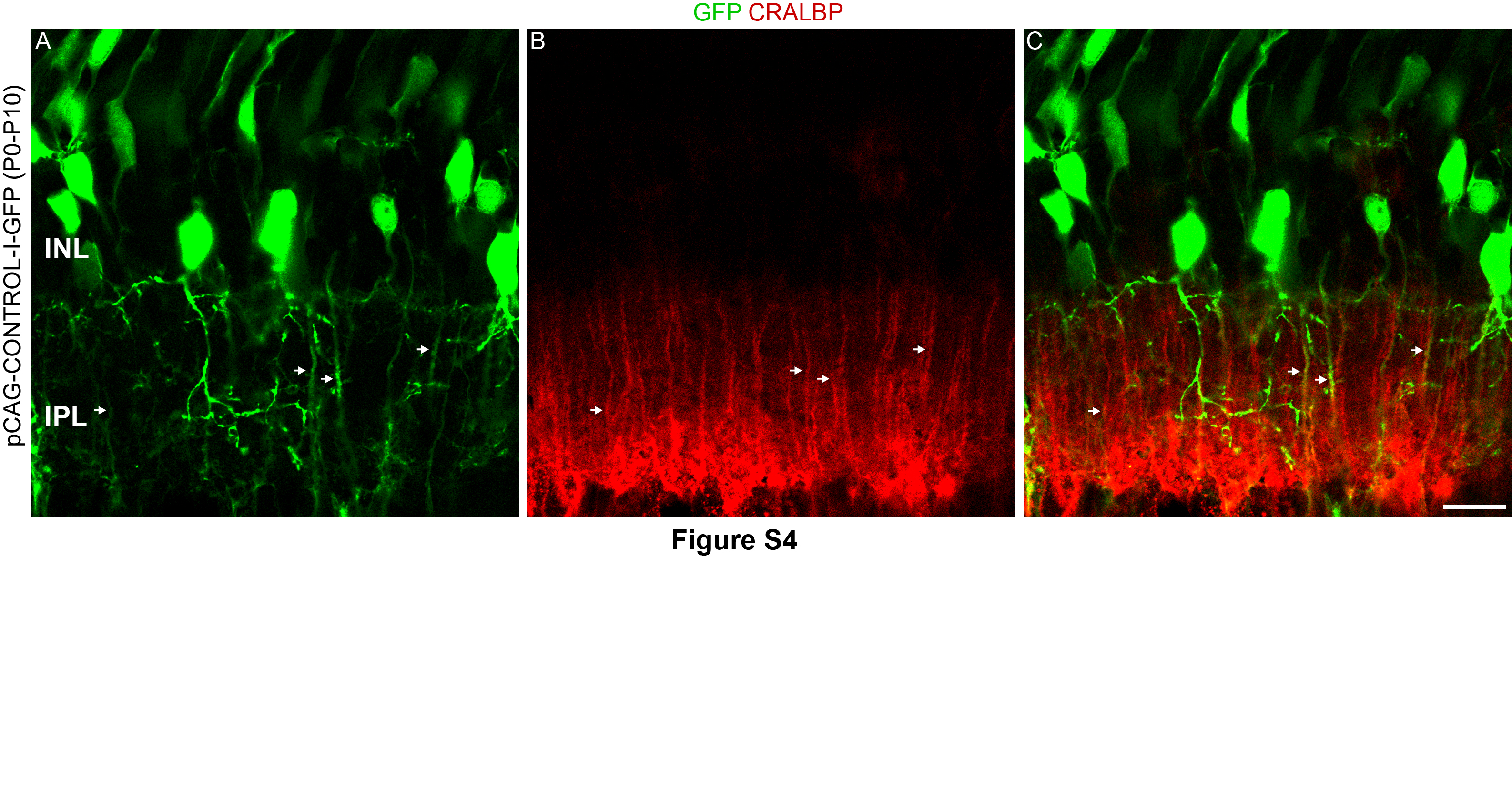

Supplement: Figure S4 — Expression of CRALBP in MGCs generated in the postnatal retina electroporated with control-I-GFP. (A–C) Coronal section of a P10 rat retina after electroporation with Control-I-GFP at P0, immunolabeled for GFP (green) and CRALBP (red). Images are single confocal Z-stacks and show the co-localization of GFP and CRALB in MGC fibers (arrows). Scale bar: 25 μm. [file Image_4.JPEG]

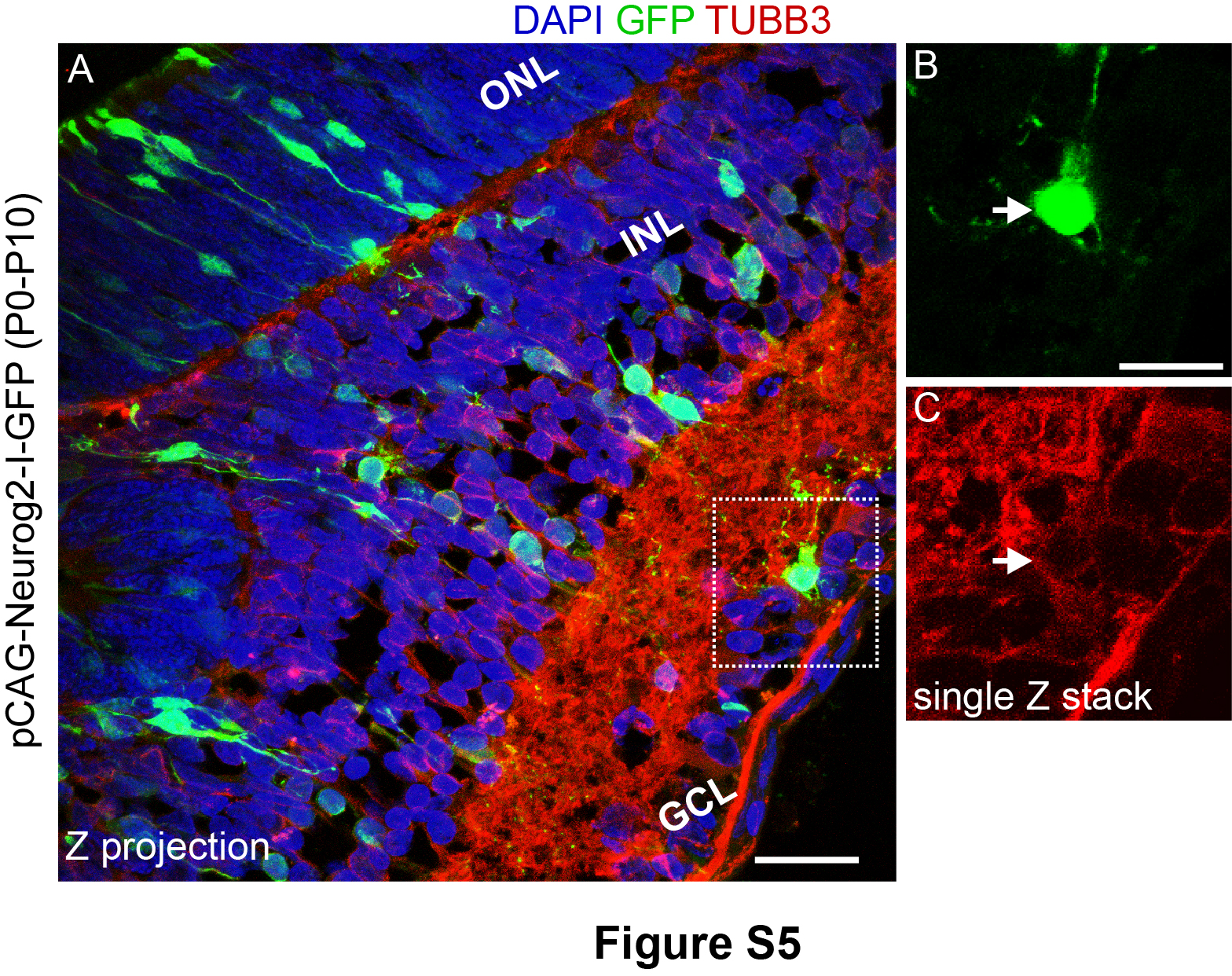

Supplement: Figure S5 — Expression of βIII-TUBULIN in RGCs generated in the postnatal retina following Neurog2-electroporation. (A) Coronal section of a P10 rat retina after electroporation with Neurog2-I-GFP at P0, immunolabeled for GFP (green) and βIII-TUBULIN (TUBB3, red). Nuclei are stained with DAPI (blue). Image is a Z-projection of 8 confocal Z-stacks. Dashed box delimits a GFP+ cell within the ganglion cell layer (GCL). (B,C) Magnification of the dashed box in A showing the co-localization of GFP and βIII-TUBULIN in a single confocal Z-stack. Scale bars: A: 50 μm; B,C: 25 μm. [file Image_5.JPEG]
